# ZILVERPASS Study: ZILVER PTX Stent versus Prosthetic Above-the-Knee Bypass Surgery in Femoropopliteal Lesions, 5-year Results

**DOI:** 10.1007/s00270-023-03549-0

**Published:** 2023-09-05

**Authors:** Michel J. Bosiers, Gianmarco De Donato, Giovanni Torsello, Pierre Galvagni Silveira, Dierk Scheinert, Pierfrancesco Veroux, Jeroen Hendriks, Lieven Maene, Koen Keirse, Tulio Navarro, Hans-Henning Eckstein, Jörg Teβarek, Alessia Giaquinta, Wouter van den Eynde, Jürgen Verbist, Joren Callaert, Koen Deloose, Marc Bosiers

**Affiliations:** 1 Department of Vascular Surgery, St. Franziskus-Hospital, Münster, Germany; 2 Department of Vascular Surgery, University Hospital Bern, University of Bern, Freiburgstrasse 18, 3010 Bern, Switzerland; 3 Department of Vascular Surgery, University of Siena, Siena, Italy; 4 Department of Vascular Surgery, UFSC University Hospital Santa Catarina, Florianópolis, Brazil; 5 Department of Angiology, University Hospital of Leipzig, Leipzig, Germany; 6 Department of Vascular Surgery, University Hospital of Catania, Catania, Italy; 7 Department of Vascular Surgery, University Hospital of Antwerp, Edegem, Belgium; 8 Department of Vascular Surgery, OLV Hospital Aalst, Aalst, Belgium; 9 Department of Vascular Surgery, RZ Heilig Hart Tienen, Tienen, Belgium; 10 Department of Vascular Surgery, Federal University of Minas Gerais, Belo Horizonte, Brazil; 11 Department of Vascular Surgery, Klinikum Rechts Der Isar, Munich, Germany; 12 Department of Vascular Surgery, St. Bonifatius Hospital Lingen, Lingen, Germany; 13 Department of Vascular Surgery, Imelda Hospital, Bonheiden, Belgium; 14 Department of Vascular Surgery, AZ St. Blasius, Dendermonde, Belgium

**Keywords:** PAD, Femoropopliteal, Drug-eluting-stent, Prosthetic bypass

## Abstract

**Purpose:**

To report the 60-month safety and effectiveness results of a multicenter, prospective, randomized controlled trial comparing the ZILVER PTX paclitaxel-eluting stent to prosthetic above-the-knee bypass for the treatment of symptomatic TransAtlantic Inter-Society Consensus (TASC) C and D femoropopliteal lesions.

**Materials and methods:**

Patients were enrolled between October 2013 and July 2017. One of the secondary outcomes was primary patency at 60 months, defined as no evidence of binary restenosis or occlusion within the target lesion or bypass graft based on a duplex ultrasound peak systolic velocity ratio < 2.4 and no clinically-driven target lesion revascularization (TLR) in endovascular cases or reintervention to restore flow in the bypass at 60 months. Survival rates after 5 years were also analyzed.

**Results:**

220 patients (mean age 68.6 ± 10.5 years; 159 men) were included and randomized to ZILVER PTX (n = 113, 51.40%) or BYPASS group (n = 107, 48.60%). The 60-month primary patency rate was 49.3% for the ZILVER PTX group versus 40.7% for the bypass group (*p* = 0.6915). Freedom from TLR was 63.8% for the ZILVER PTX group versus 52.8% for the bypass group (*p* = 0.2637). At 5 years, no significant difference in survival rate could be seen between the ZILVER PTX and the bypass group (69.1% vs. 71% respectively, *p* = 0.5503).

**Conclusion:**

Even at 5 years, non-inferior safety and effectiveness results of the ZILVER PTX could be seen. These findings confirmed that the use of ZILVER PTX stents can be considered as a valid alternative for bypass surgery when treating long and complex femoropopliteal lesions.

**Graphical Abstract:**

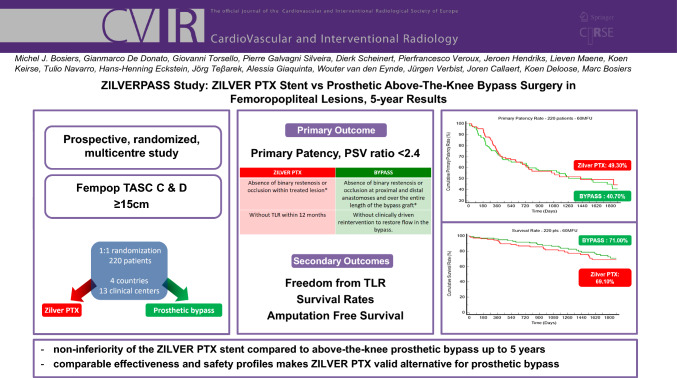

## Introduction

The prevalence of peripheral arterial disease (PAD) is on the rise, affecting over 202 million individuals worldwide [1]. Relevant risk factors contributing to this elevated prevalence include smoking, diabetes mellitus, hypertension, hypercholesterolemia age and obesity [1]. PAD can present itself asymptomatically or symptomatically by intermittent claudication (IC) or critical limb threatening ischemia (CLTI). Conservative management of PAD encompasses smoking cessation, exercise and management of other risk factors associated with the disease. However, a revascularization procedure becomes necessary for patients with disabling IC, CLTI or when conservative measures prove inadequate.

The TransAtlantic Inter-Society Consensus II (TASC) recommendations for the treatment of PAD in the femoropopliteal segment are well established in the vascular community. Where for TASC A and B lesions, an endovascular approach is recommended, for more challenging TASC C and D lesions, surgical bypass treatment is recommended [2]. However, these guidelines from 2007 were based on older endovascular techniques. Even current guidelines recommend endovascular treatment for femoropopliteal lesions < 25 cm and advancements in techniques and stent-designs appear to enhance long-term patency [3].

Earlier studies investigating the use of a drug-eluting ZILVER PTX stent (Cook Medical, Bloomington, IN, USA) in femoropopliteal TASC C and D lesions have shown good results. In a subgroup analysis of a prospective, multicenter study, a 12-month primary patency rate of 77.6% was observed when treating complex lesions with a mean lesion length of 226.10 mm [4]. In a prospective, multicenter, post-market surveillance study in Japan, the ZILVER-PTX stent was employed in 1075 lesions with a mean lesion length of 147 mm. The 12-month primary patency rate was 86.4% [5].

Based on these results, a randomized controlled trial was initiated to ascertain whether the ZILVER PTX paclitaxel-eluting stent demonstrated noninferiority to surgical above-the-knee prosthetic bypass in TASC C and D femoropopliteal lesions. Results at 12- and 36-months, along with an economic analysis, were previously published [6, 7]. This paper now presents the 5-year results from this study.

## Materials and Methods

### Study Design

The study design has been previously described [6]. In summary, the ZILVERPASS-trial was a global, prospective, randomized, controlled, noninferiority study. Patients were enrolled between October 2013 and July 2017 at 13 clinical sites in Belgium, Germany, Italy and Brazil. The study was conducted in accordance with ISO 14155. The local ethical committees at the participating sites approved the study protocol, and all patients provided written informed consent prior to undergoing any study-related procedures. The trial was registered on the National Institutes of Health website (*ClinicalTrials.gov* identifier NCT01952457).

The study designers aimed to ensure consistent treatment in the control arm. To achieve this, they selected one type of bypass graft based on existing literature that compared prosthetic to venous grafts for above-the-knee femoropopliteal bypass procedures [8–11]. Because there was no significant difference in the 1-year primary and 3-year secondary patency rates, the study designers decided to use prosthetic bypass grafts as the comparising group. Additionally, some of the study investigators noted that prosthetic bypass was the standard of care for above-the-knee lesions at their medical facility.

Table 1 gives an overview of the inclusion and exclusion criteria. In brief, patients with moderate to severe intermittent claudication or chronic limb-threatening ischemia (CLTI) with rest pain or minor tissue loss (Rutherford categories 2 to 5) presenting with a long (≥ 15 cm) stenotic or occlusive de novo lesion (TASC C and D) in the femoropopliteal arteries suitable for both endovascular therapy and bypass surgery were included in this study.

**Table 1:** Inclusion and Exclusion Criteria

Inclusion Criteria—General	
1	Patient presenting with lifestyle-limiting claudication, rest pain or minor tissue loss (Rutherford category 2 to 5)
2	Patient is willing to comply with specified follow-up evaluations at the specified times
3	Patient is > 18 years old
4	Patient understands the nature of the procedure and provides written informed consent, prior to enrollment in the study
5	Patient has a projected life expectancy of at least 24 months
6	Noninvasive lower extremity arterial studies (resting or exercise) demonstrate ankle-brachial index ≤ 0.8
7	Patient is eligible for treatment with the Zilver PTX paclitaxel-eluting stent (Cook) or with surgical above-the-knee bypass placement
8	Male, infertile female, or female of child bearing potential practicing an acceptable method of birth control with a negative pregnancy test within 7 days prior to study procedure
Inclusion Criteria—Angiographic	
1	Stenotic or occlusive de novo lesion located in the femoropopliteal arteries, suitable for endovascular therapy and for bypass surgery
2	Total target lesion length is at least 15 cm
3	Minimum of 1.0 cm of healthy vessel (non-stenotic) both proximal and distal to the treatment area
4	P2 and P3 are patent and there is angiographic evidence of at least 1 vessel runoff to the foot, that does not require intervention (< 50% stenotic)
5	Target vessel diameter visually estimated to be > 4 mm and < 9 mm at the proximal and distal treatment segments within the SFA
Exclusion Criteria	
1	Untreated flow-limiting aortoiliac stenotic disease
2	Any previous surgery and endovascular procedure in the target vessel
3	Severe ipsilateral common/deep femoral disease requiring surgical reintervention
4	Perioperative unsuccessful ipsilateral percutaneous vascular procedure to treat inflow disease just prior to enrollment
5	Femoral or popliteal aneurysm located at the target vessel
6	Non-atherosclerotic disease resulting in occlusion (eg, embolism, Buerger’s disease, vasculitis)
7	No patent tibial arteries (> 50% stenosis)
8	Prior ipsilateral femoral artery bypass
9	Severe medical comorbidities (untreated CAD/CHF, severe COPD, metastatic malignancy, dementia, etc.) or other medical condition that would preclude compliance with the study protocol or 2-year life expectancy
10	Serum creatinine > 2.5 mg/dL within 45 days prior to study procedure unless the subject is currently on dialysis
11	Major amputation (above the transmetatarsal) in the study or non-study limb
12	Any previously known coagulation disorder, including hypercoagulability
13	Contraindication to anticoagulation or antiplatelet therapy
14	Known allergies to stent components (nickel-titanium, paclitaxel, etc.) or bypass graft components (Dacron, ePTFE, etc.)
15	Known allergy to contrast media that cannot be adequately premedicated prior to the study procedure
16	Currently participating in another clinical research trial
17	Angiographic evidence of intra-arterial thrombus or atheroembolism from inflow treatment
18	Any planned surgical intervention/procedure within 30 days of the study procedure
19	Target lesion access in the Zilver PTX stent arm not performed by transfemoral approach

CAD, coronary artery disease; CHF, congestive heart failure; COPD, chronic obstructive pulmonary disease; ePTFE, expanded polytetrafluoroethylene; SFA, superficial femoral artery

To determine if the effect of treatment with the ZILVER PTX paclitaxel-eluting stent was not inferior to the effect of treatment with surgical bypass, a sample size of 220 patients was calculated, assuming the 1-year primary patency rates for the bypass arm and the ZILVER PTX arm were 70.00% and 80.00%, respectively. The null hypothesis was that the primary patency rate for the ZILVER PTX arm was 6.00% lower than the primary patency rate for the bypass arm; the study had a power of 86.50% to reject the null hypothesis.

### Study Devices

The ZILVER PTX stent is a self-expanding nitinol stent coated with polymer-free paclitaxel (3 µg/mm^2^ dose density) and designed to provide support while maintaining flexibility in the vessel upon deployment. The device is approved by the Conformité Européenne for use in patients with atherosclerotic disease of the above-the-knee femoropopliteal arteries.

The device used for the control arm was a prosthetic bypass from the common femoral artery to the above-the-knee popliteal artery (P1 segment). The type of prosthetic bypass (Dacron or expanded polytetrafluoroethylene) was at the physician’s discretion.

### Randomization and Masking

Using a computer-generated list for each site, permuted block randomization was applied with block sizes of 8 patients assigned in a 1:1 fashion to either endovascular treatment with the ZILVER PTX stent or BYPASS surgery with synthetic graft. Each site received closed envelopes that were opened only after the patient signed the informed consent. Once a treatment was assigned, crossover was not permitted. Patients assigned to the ZILVER PTX group were considered enrolled when successful lesion passage was achieved and diagnostic angiography confirmed that all angiographic inclusion criteria were met. Patients assigned to the BYPASS group were considered enrolled when they were treated as intended.

### Procedures

Standard procedures were followed based on the instructions for use for the devices. Intraoperative heparinization (5000 units) was applied in both treatment groups. The only pre-treatment allowed prior to placement of the ZILVER PTX stent was standard balloon angioplasty. The use of pre- and postdilation was recommended but at the physician’s discretion. Angiography immediately after the endovascular intervention was required to evaluate the postoperative lesion. Following treatment, antiplatelet therapy consisting of clopidogrel for at least 60 days and lifelong aspirin therapy was routinely prescribed. Physical examination was performed prior to discharge. The follow-up data collection points were 1, 6, 12, 24, 36, and 60 months, with unplanned or interim visits as need for recurrent symptoms or complications. Follow-up visits included ABI measurements, Rutherford category assessment, and duplex ultrasound examination.

### Outcome Definitions

The study was extended to a 5-year follow-up for the primary outcome measure: primary patency, defined as no evidence of binary restenosis or occlusion within the target lesion based on a duplex-derived peak systolic velocity ratio (PSVR) < 2.4 and no clinically-driven target lesion revascularization (TLR). In the bypass arm, patency was defined as no evidence of binary restenosis or occlusion at the anastomoses or over the entire length of the bypass graft based on a PSVR < 2.4 and no clinically-driven reintervention to restore flow in the bypass.

Secondary outcomes, which were analyzed at 5-year follow-up, included freedom from TLR-rate, overall survival rate and freedom from amputation rate. The latter was stratified to claudicants and CLTI patients in both groups.

### Statistical Analysis

Continuous data are presented as the mean ± standard deviation (range); categorical data are given as the number (percentage). The primary patency rates were estimated using Kaplan–Meier survival analysis; the estimates are given with the 95% confidence interval. Curves were compared using the log-rank test. Sub analyses were performed to evaluate differences in primary patency based on baseline characteristics (smoking history, hypertension, diabetes, renal insufficiency, obesity, hypercholesterolemia), symptom status (claudication vs. CLTI), and presence of total occlusions. The threshold of statistical significance was *p* < 0.05. All statistical analyses were completed with IBM SPSS Statistical Software for Windows (version 22.0; IBM Corporation, Armonk, New York).

## Results

A total of 220 patients (mean age 68.6 ± 10.5 years; 159 men) were enrolled and randomized to the ZILVER PTX treatment group (n = 113, 51.40%) or the BYPASS treatment group (n = 107, 48.60%). Patient characteristics are presented in Table 2. The most prominent risk factor was nicotine abuse (n = 164, 74.50%), followed by hypertension (n = 161, 73.20%) and hypercholesterolemia (n = 127, 57.70%). The majority of the patients were claudicants (n = 139, 63.20%), but the bypass patients had significantly more CLTI, as well as hypertension, hypercholesterolemia, and obesity, despite randomization. Most of the lesions (Table 3) were occlusions (n = 208, 94.50%). Overall mean lesion length was 247.10 ± 69.30 mm and did not differ between the groups. All endoluminal procedures were successful, and no crossover was observed.

**Table 2:** Baseline Patient Characteristics^a^

	Total (n = 220)	ZILVER PTX (n = 113)	Bypass (n = 107)	*p*
Age, y	68.6 ± 10.5	69.6 ± 10.8	67.6 ± 10.1	0.305
Men	159 (72.3)	78 (69.0)	81 (75.7)	0.267
Claudication	139 (63.2)	80 (70.8)	59 (55.1)	0.016
CLTI	81 (36.8)	33 (29.2)	48 (44.9)	
Smoking history	164 (74.5)	78 (69.0)	86 (80.4)	0.053
Hypertension	161 (73.2)	74 (65.5)	87 (81.3)	0.008
Diabetes	65 (29.5)	31 (27.4)	34 (31.8)	0.480
Coronary artery disease	58 (26.4)	26 (23.0)	32 (29.9)	0.246
Cerebrovascular disease	14 (6.4)	8 (7.1)	6 (5.6)	0.655
Renal insufficiency	24 (10.9)	11 (9.7)	13 (12.1)	0.566
Obesity	30 (13.6)	10 (8.8)	20 (18.7)	0.033
Hypercholesterolemia	127 (57.7)	57 (50.4)	70 (65.4)	0.025

CLTI, chronic limb-threatening ischemia

^a^Continuous data are presented as the mean ± standard deviation; categorical data are given as the number (percentage)

**Table 3:** Lesion and Procedure Characteristics^a^

	Total (n = 220)	ZILVER PTX (n = 113)	Bypass (n = 107)	*p*
Study limb				
Left	114 (51.8)	61 (54.0)	53 (49.5)	0.509
Right	106 (48.2)	52 (46.0)	54 (50.6)	
Lesion type				
Stenosis	12 (5.5)	9 (8.0)	3 (2.8)	0.092
Occlusion	208 (94.5)	104 (92.0)	104 (97.2)	
TASC C	12	9	3	
TASC D	208	104	104	
Lesion length, mm	247.1 ± 69.3 (100–500)	241.7 ± 63.3 (120–500)	252.9 ± 74.9 (100–400)	0.104
Proximal RVD, mm	5.9 ± 0.7 (4.0–8.0)	5.7 ± 0.7 (4.4–8.0)	6.1 ± 0.8 (4.0– 8.0)	0.320
Procedure time, min	90.5 ± 44.8 (17–240)	59.6 ± 22.7 (17–135)	123.1 ± 38.9 (53–240)	< 0.001
Hospital stay, d	5.3 ± 5.7 (0–34)	2.5 ± 3.5 (0–20)	8.1 ± 6.0 (1–34)	< 0.001
Bypass material (Dacron/PTFE)	NA	NA	42/65	

NA, not applicable; PTFE, polytetrafluoroethylene; RVD, reference vessel diameter; TASC, TransAtlantic Inter-Society Consensus

^a^Continuous data are presented as the mean ± standard deviation (range); categorical data are given as the number (percentage)

**Fig. 1:**
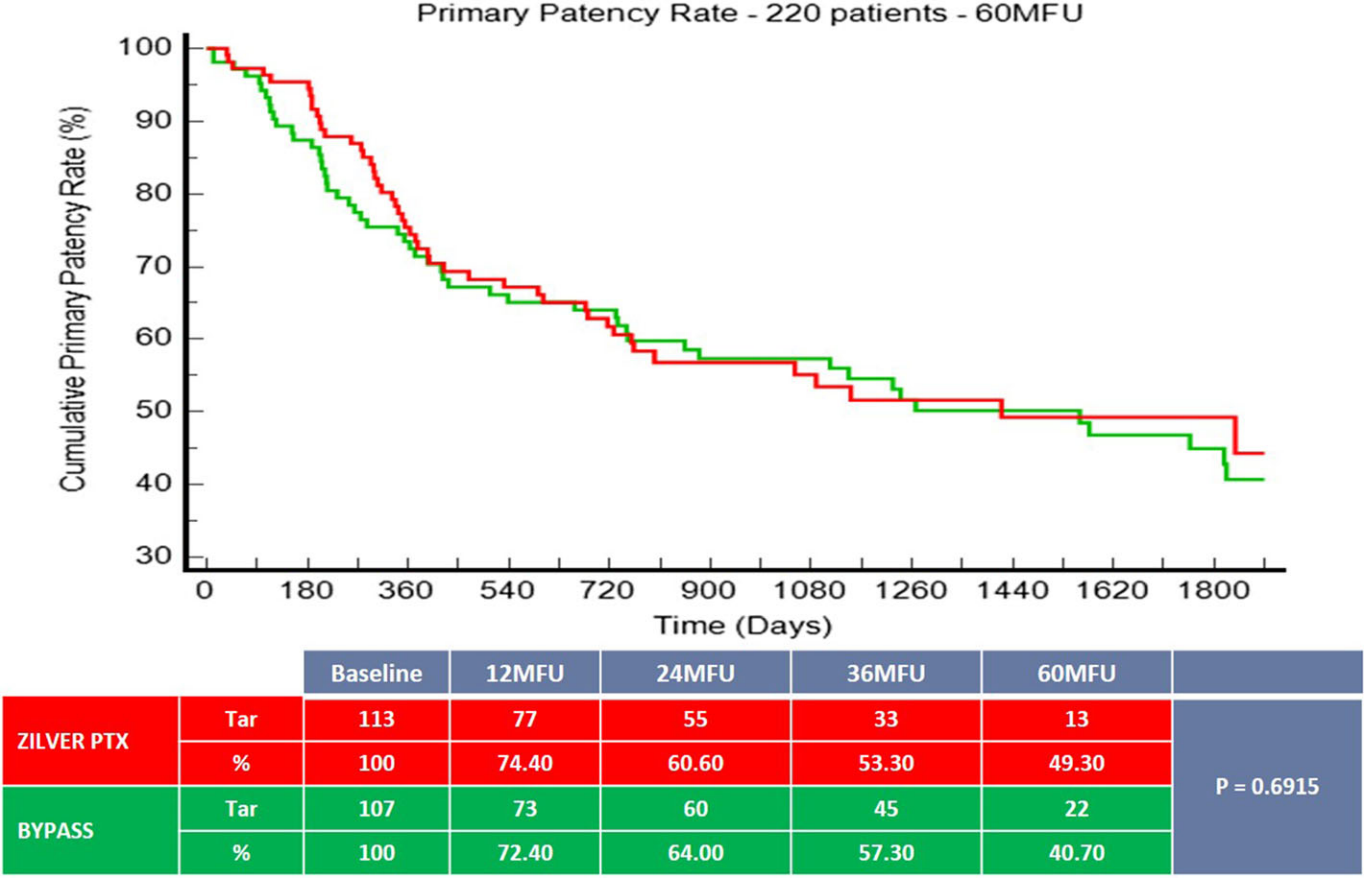
Primary patency rate

The 1-, 3- and 5-year primary patency rate (Fig. 1) was 74.4%, 53.3% and 49.30% (95% CI 53.60% to 45.00%) respectively for the ZILVER PTX group versus 72.4%, 57.3% and 40.70% (95% CI 46.50% to 34.90%) respectively for the BYPASS group (*p* = 0.6915).

**Fig. 2:**
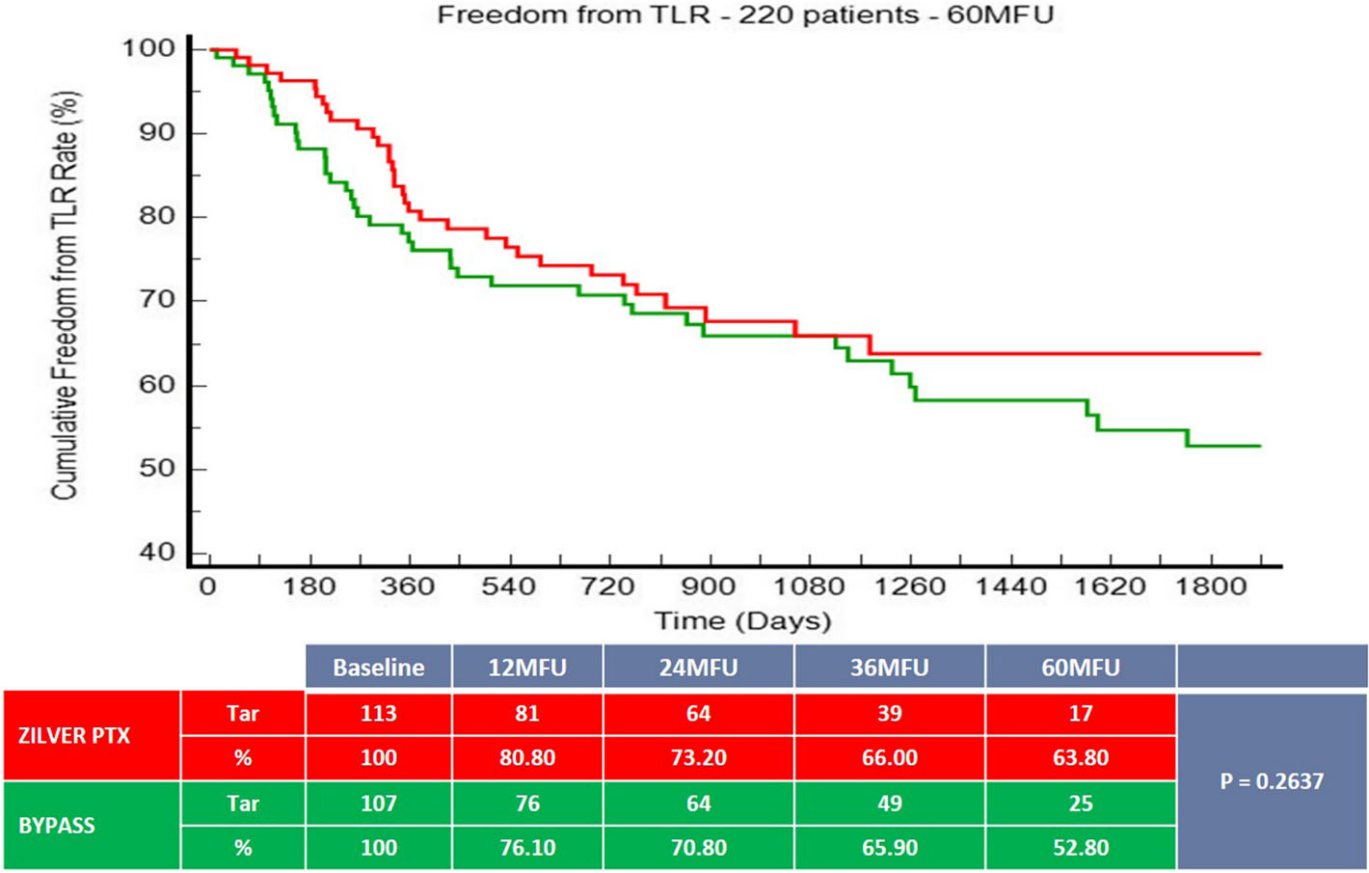
Freedom from TLR

Freedom from TLR at 1-, 3- and 5-year (Fig. 2) was 80.8%, 66% and 63.80% (95% CI 71.00% to 56.60%) for the ZILVER PTX group versus 76.1%, 65.9% and 52.80% (95% CI 57.90% to 47.70%) for the BYPASS group (*p* = 0.2637).

**Fig. 3:**
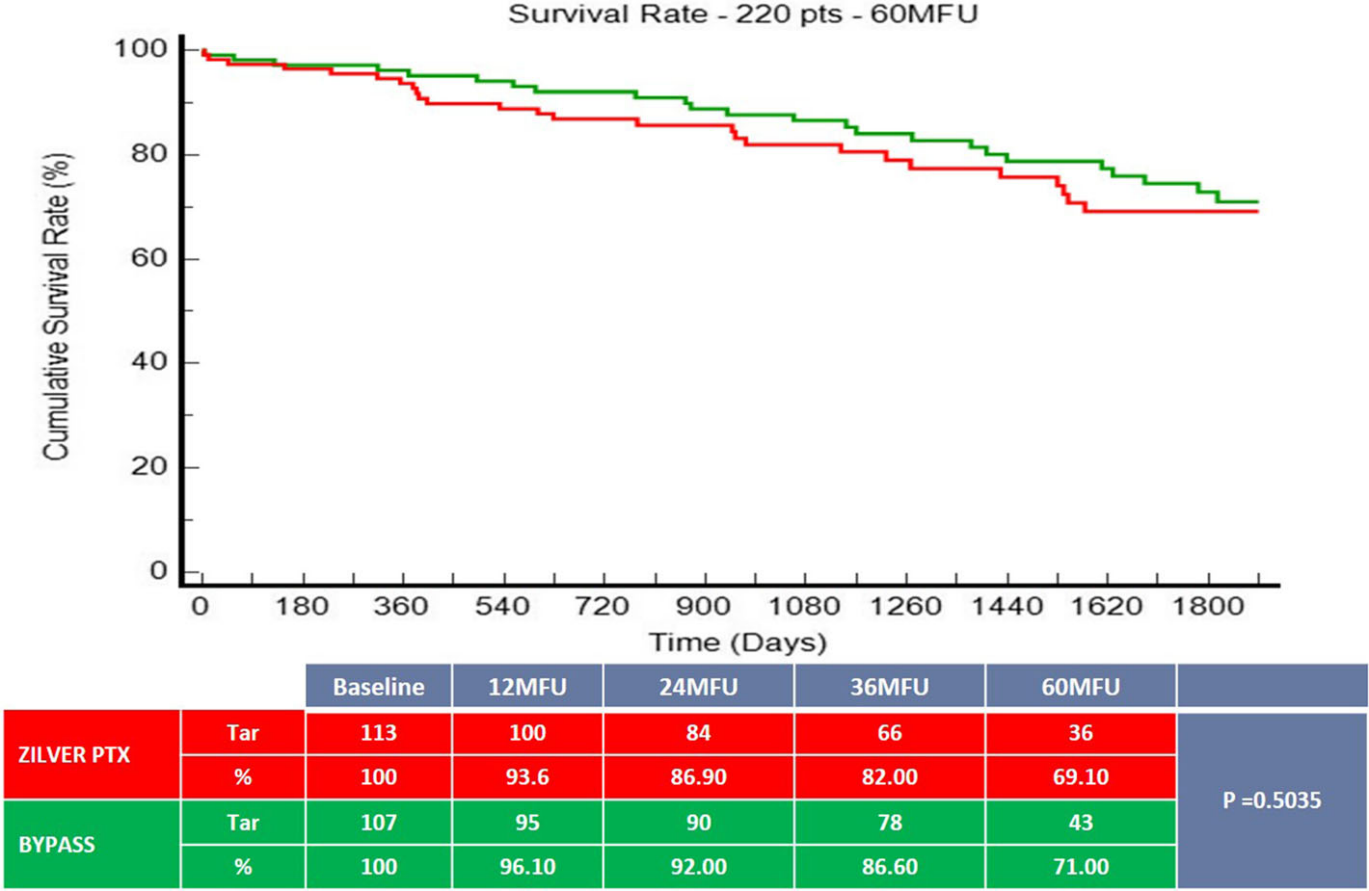
Survival rate

There was no significant difference (*p* = 0.5503) in survival rate at 5-year between the ZILVER PTX group 69.10%, (95% CI to 74.30% to 63.90%) and the BYPASS group 71.00% (95% CI 76.80% to 65.20%) (Fig. 3). None of the deaths was categorized as device- and/or procedure-related. There was a plethora of reasons for patients’ death, such as myocardial infarction, pneumonia, cancer, etc.

**Fig. 4:**
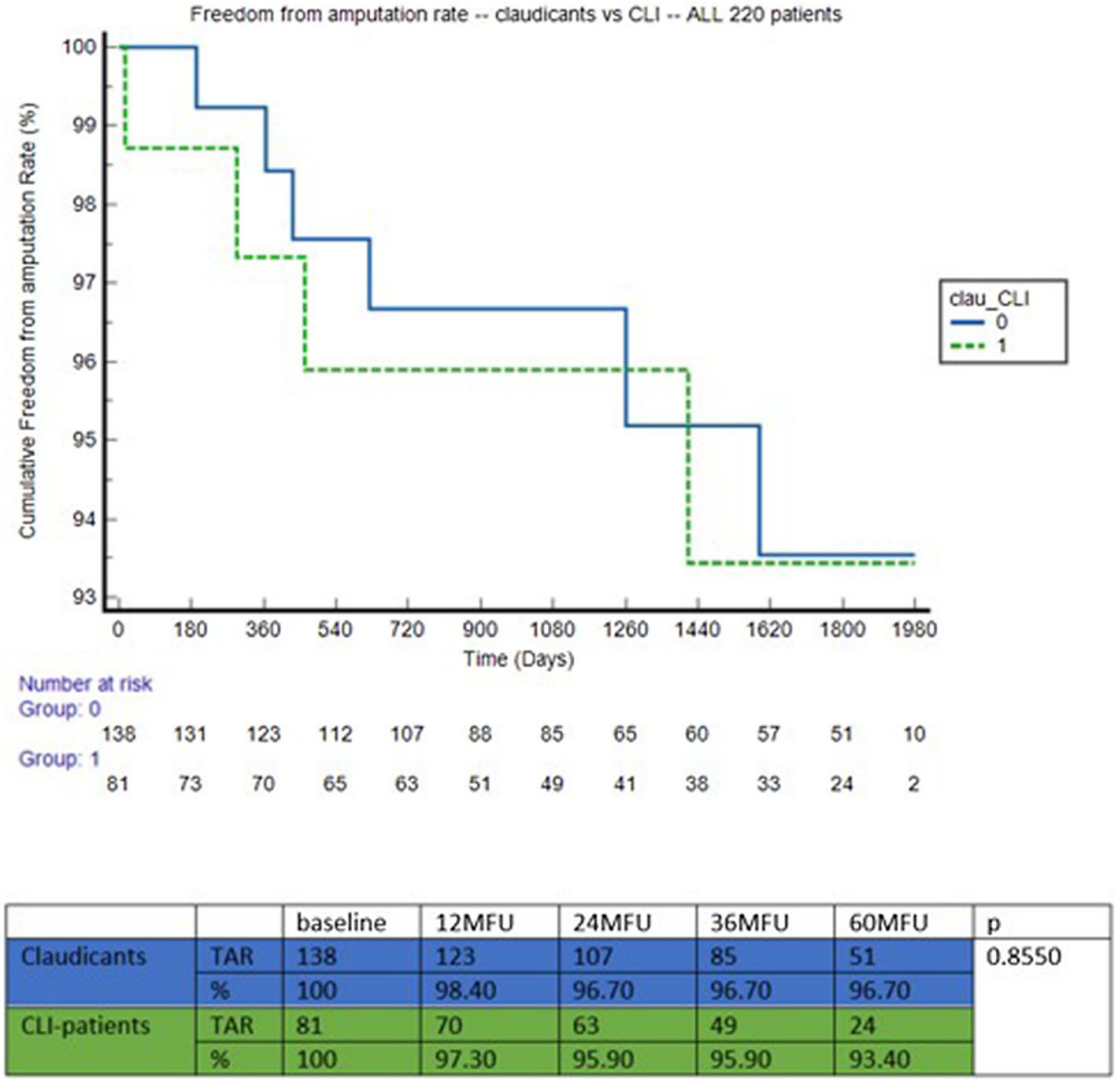
Freedom from amputation rate—claudicants versus CLTI

**Fig. 5:**
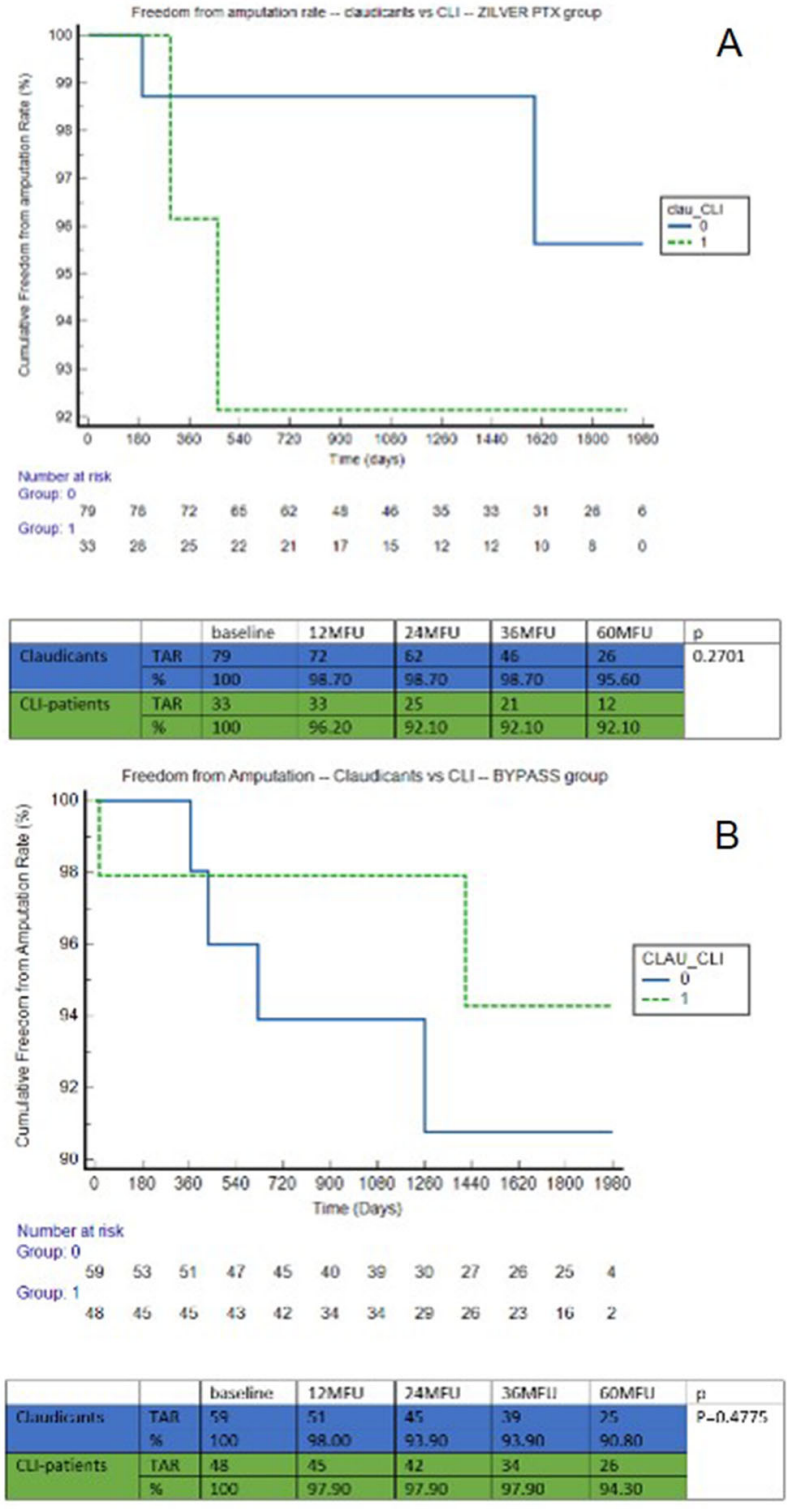
**a** Freedom from amputation rate—claudicants versus CLTI—ZILVER PTX, **b** Freedom from amputation rate—claudicants versus CLTI—BYPASS

Freedom from amputation rate at 5-year was 94.60% in the ZILVER PTX group versus 92.50% in the BYPASS group (*p* = 0.5818). We found no significant difference when performing a sub analysis for claudicants versus CLTI patients (*p* = 0.8550) (Fig. 4). When looking at both groups individually, no significant difference in freedom from amputation rate could be observed in both ZILVER PTX (*p* = 0.2701) and BYPASS group (*p* = 0.4775) (Fig. 5) in terms of claudicants versus CLTI.

Multi-variate analyses were performed for tobacco use, hypertension status, diabetes status, history of PAD, history of coronary artery disease, history of cerebrovascular disease, renal insufficiency status, obesity status, hypercholesterolemia status, claudicants versus CLTI patients and stenosed versus occluded lesions, both in the BYPASS and the ZILVER PTX group. At 5-year follow-up there were no differences seen between these groups in terms of primary patency. An overview of the results of the multi-variate analysis can be found in Table 4.

**Table 4:** Multi-variate analysis: 60-month primary patency

	No smoking history	Smoking history	
BYPASS	34.40%	42.50%	0.2589
ZILVER PTX	46.60%	50.60%	0.2128
	No hypertension	Hypertension	
BYPASS	48.10%	40.30%	0.3024
ZILVER PTX	44.80%	56.00%	0.5997
	No diabetes	Diabetes	
BYPASS	44.40%	40.20%	0.6697
ZILVER PTX	50.00%	50.50%	0.9797
	No history of PAD	History of PAD	
BYPASS	43.00%	39.80%	0.9295
ZILVER PTX	51.60%	29.40%	0.9576
	No history of CAD	History of CAD	
BYPASS	44.60%	32.20%	0.5348
ZILVER PTX	49.90%	51.00%	0.4149
	No history of cerebrovascular disease	History of cerebrovascular disease	
BYPASS	43.10%	0.00%	0.2831
ZILVER PTX	47.00%	75.00%	0.5795
	No renal insufficiency	Renal insufficiency	
BYPASS	37.60%	68.70%	0.1301
ZILVER PTX	48.70%	57.90%	0.2658
	No obesity	Obesity	
BYPASS	42.30%	29.90%	0.8904
ZILVER PTX	48.70%	58.30%	0.4509
	No hypercholesterolemia	Hypercholesterolemia	
BYPASS	38.10%	41.10%	0.9832
ZILVER PTX	54.40%	45.10%	0.6162
	Claudicants (RF 2,3)	CLI patients (RF 4,5)	
BYPASS	41.90%	39.30%	0.7388
ZILVER PTX	53.50%	39.10%	0.4267
	Stenosed lesion	Occluded lesion	
BYPASS	100.00%	41.00%	0.6841
ZILVER PTX	75.00%	47.40%	6.3136

## Discussion

We present long-term data concerning the treatment of long (almost 25 cm) and complex (94.55% occlusion) femoropopliteal lesions using drug-eluting stents (ZILVER PTX) in a controlled manner. This study demonstrates, at the very least, the non-inferiority of ZILVER PTX compared to above-the-knee prosthetic bypass. A primary patency rate of 49.3% in the ZILVER PTX group after 60 months is off course lower than the 64.9% after 5 years reported in the ZILVER PTX randomized trial [12], which compared this drug-eluting stent with PTA plus provisional stenting. One of the primary reasons for this difference is the lesion complexity. The latter study involved lesions with an average lesion length of approximately 66.4 mm and occlusion in only 32.8%.

Recently, 2 multicenter, randomized controlled trials were published: the Best Endovascular versus Best Surgical Therapy for Patients with Critical Limb Ischemia (BEST-CLI) [13] and Bypass versus Angioplasty in Severe Ischemia of the Leg-2 (BASIL-2) [14]. Both trials aimed to determine the most effective treatment modality for patients with CLTI. The BEST-CLI authors concluded that patients with an adequate great saphenous vein (GSV), eligible for both strategies, would benefit more from open bypass surgery. However, in cases were a suitable vein was unavailable, there was no significant difference in outcomes between open bypass surgery and endovascular therapy. It is important to note that 21.6% of randomized patients in the BEST-CLI study lacked an adequate GSV. For various obvious reasons, the BEST-CLI trial and the ZILVERPASS trial cannot be directly compared. Nevertheless, the findings suggest the same: the non-inferiority of endovascular therapy versus prosthetic bypass. BASIL-2 had a different primary outcome, namely amputation-free survival. The authors identified a higher risk of major amputation or death in the vein bypass group compared to the endovascular group, primarily driven by an increased death rate. While direct comparison with the ZILVERPASS trial is challenging due to differences in treatment (infrapopliteal vs. above-the-knee), no significant difference in the freedom from amputation rate after 60 months was observed between claudicants and CLTI patients in the current trial, even when analyzed separately. Furthermore, regarding deaths, a significant difference in DES versus prosthetic above-the-knee bypass could not be discerned in the ZILVERPASS trial.

Evidence also exists for the long-term outcomes of bypass surgery. In a prospective, randomized, multicenter study by Midy et al. [11], the efficacy of prosthetic and autologous vein for above-the-knee femoropopliteal bypasses was compared. The 5-year secondary patency rates were 84.6% and 70.8% for the prosthetic group and autologous vein group, respectively, leading to the conclusion that there was no significant difference between the treatment modalities. Primary patency data was not reported. A recent review highlighted the lack of high-quality randomized data for patients with IC receiving above-the-knee bypass [15]. However, the authors concluded that, when a good saphenous vein is present, venous bypass remains the preferred therapy when a bypass is necessary. Many of these trials used varying definitions to assess primary and secondary patency. In the current study, a consistent definition for both groups was used regarding primary patency- defined as the absence of binary restenosis (PSVR < 2.4) and not solely flow through the bypass.

In 2018, Katsanos et al. [16] shocked the vascular community by reporting a mortality signal indicating an increased mortality when using paclitaxel coated devices in the femoropopliteal segment. Since then, a plethora of evidence has been published, refuting the existence of such as signal [17]. In July 2023, the FDA released a statement indicating that based on the updated data and analyses, there is no substantial evidence to support the previously suggested excess mortality [18]. In our study, we found no significant difference in the 5-year survival rate between the ZILVER PTX group (69.10%) and the BYPASS group (71%), further supporting the safety of paclitaxel coated devices.

Recently, alongside the 3-year data from the ZILVERPASS trial, an economic analysis for Germany and the USA was published [7], taking procedural-, hospitalization- and reintervention costs into account, from a payor’s perspective.. A clear cost–benefit in favor of the ZILVER PTX was seen in both countries at 3 years: €9446 per patient in the BYPASS group versus €5755 per patient in the ZILVER PTX group in Germany and $26,373 per patient in the BYPASS group versus $19,186 per patient in the ZILVER PTX group in the USA.

This economic finding, coupled with the non-inferiority of Zilver PTX compared to prosthetic above-the-knee bypass at 5 years, underscores the role of an endovascular strategy with DES for TASC II C and D lesions.

### Limitations

The primary limitation of this study is the utilization of prosthetic bypass grafts for the control arm. Although venous bypass remains the recommended approach for above-the-knee bypass, prosthetic bypass was selected for 2 reasons. Firstly, within the participating centers, there was a prevailing practice to use a prosthetic bypass for above-the-knee procedures, aimed at preserving the saphenous vein for potential future below-the-knee bypass surgeries. Secondly, as previously mentioned, certain studies have indicated comparable patency outcomes between above-the-knee prosthetic and venous conduits, while high-quality evidence is lacking. An additional limitation pertains to the variation in patient population between the 2 study groups, despite randomization. A higher proportion of patients in the BYPASS group exhibited conditions such as hypertension hypercholesterolemia, obesity and CLTI. However, the multivariate analysis illustrated no discernable distinction in terms of primary patency rates. Moreover, a further limitation is the requirement for a 1 cm healthy vessel in the proximal SFA, which might restrict the applicability of this trial. Another limitation to consider is that patients were not included in the endovascular treatment group unless the lesion was effectively crossed. Nevertheless, it is worth noting that all lesions were successfully crossed and no crossover from the ZILVER PTX group to the BYPASS group occurred.

## Conclusion

These final 5-year results unequivocally establish the non-inferiority of the ZILVER PTX stent compared to above-the-knee prosthetic bypass surgery, underscoring their comparable effectiveness and safety profiles. The ZILVERPASS trial affirms that the ZILVER PTX stent treatment can be regarded as a valid alternative for bypass surgery in cases involving long and complex femoropopliteal lesions.
